# Real-world growth outcomes of vosoritide in children with achondroplasia: a single-center cohort study

**DOI:** 10.1210/jendso/bvag072

**Published:** 2026-03-28

**Authors:** Ravit Regev, Yarden Waksman, Eyal Cohen-Sela, Avivit Brener, Nechama Dechovich, Maya Gal, Ophir Borger, Leonid Zeitlin, Yael Lebenthal

**Affiliations:** Institute of Pediatric Endocrinology, Diabetes and Metabolism, Dana-Dwek Children's Hospital, Tel Aviv Sourasky Medical Center, Tel Aviv 6423906, Israel, affiliated to The Gray Faculty of Medical & Health Sciences, Tel Aviv University, Tel Aviv, Israel; Institute of Pediatric Endocrinology, Diabetes and Metabolism, Dana-Dwek Children's Hospital, Tel Aviv Sourasky Medical Center, Tel Aviv 6423906, Israel, affiliated to The Gray Faculty of Medical & Health Sciences, Tel Aviv University, Tel Aviv, Israel; Institute of Pediatric Endocrinology, Diabetes and Metabolism, Dana-Dwek Children's Hospital, Tel Aviv Sourasky Medical Center, Tel Aviv 6423906, Israel, affiliated to The Gray Faculty of Medical & Health Sciences, Tel Aviv University, Tel Aviv, Israel; Institute of Pediatric Endocrinology, Diabetes and Metabolism, Dana-Dwek Children's Hospital, Tel Aviv Sourasky Medical Center, Tel Aviv 6423906, Israel, affiliated to The Gray Faculty of Medical & Health Sciences, Tel Aviv University, Tel Aviv, Israel; Institute of Pediatric Endocrinology, Diabetes and Metabolism, Dana-Dwek Children's Hospital, Tel Aviv Sourasky Medical Center, Tel Aviv 6423906, Israel, affiliated to The Gray Faculty of Medical & Health Sciences, Tel Aviv University, Tel Aviv, Israel; Department of Occupational Therapy, The Gray Faculty of Medical & Health Sciences, Tel Aviv University, Tel Aviv 6997801, Israel; Institute of Pediatric Endocrinology, Diabetes and Metabolism, Dana-Dwek Children's Hospital, Tel Aviv Sourasky Medical Center, Tel Aviv 6423906, Israel, affiliated to The Gray Faculty of Medical & Health Sciences, Tel Aviv University, Tel Aviv, Israel; The Nutrition & Dietetics Unit, Tel Aviv Sourasky Medical Center, Tel Aviv 6423906, Israel; Pediatric Orthopedic Department, Dana-Dwek Children's Hospital, Tel Aviv 6423906, Israel; Institute of Pediatric Endocrinology, Diabetes and Metabolism, Dana-Dwek Children's Hospital, Tel Aviv Sourasky Medical Center, Tel Aviv 6423906, Israel, affiliated to The Gray Faculty of Medical & Health Sciences, Tel Aviv University, Tel Aviv, Israel

**Keywords:** achondroplasia, body proportions, C-type natriuretic peptide (CNP) analog, growth-promoting therapy, skeletal dysplasia, vosoritide

## Abstract

**Context:**

Vosoritide, a C-type natriuretic peptide analogue, is the first approved therapy targeting the pathophysiology of achondroplasia. While clinical trials demonstrate efficacy, individuals with prior orthopedic surgeries are typically excluded, and real-world data remain limited.

**Objective:**

To evaluate real-world growth outcomes of vosoritide therapy in children with achondroplasia, including those with prior surgical interventions.

**Methods:**

This retrospective analysis included 25 children with achondroplasia (17 boys, age range 2.9-14.3 years) treated with vosoritide for a mean duration of 12.7 months at a multidisciplinary center. Z-scores were calculated using an AI-assisted growth assessment tool previously validated for children with achondroplasia. Δ z-scores for height, sitting height, arm span, and body mass index (BMI) were calculated from baseline to follow-up. Multiple linear regression analyses assessed predictors of response.

**Results:**

Mean height z-scores improved from −0.62 ± 1.09 to −0.24 ± 1.20 (*P* < .001). Mean arm span z-score increased from −1.28 ± 0.93 to -0.96 ± 0.91 (*P* = .007). Sitting height z-scores showed a non-significant trend toward improvement; BMI z-scores remained stable. Mean Δ height and Δ arm span z-score gains were +0.38 ± 0.45 and +0.32 ± 0.48, respectively. A subgroup analysis of 8 patients with vs 17 without prior limb-lengthening surgery showed no significant differences. Regression analyses adjusted for sex, age, BMI z-scores, and surgical history were likely underpowered to detect meaningful predictors of response. There were no serious treatment-related adverse events.

**Conclusion:**

Vosoritide therapy enhanced linear and appendicular growth in a real-world cohort of children with achondroplasia, including post-surgical patients. These findings align with clinical trial data and support the therapy's applicability across diverse clinical profiles.

**What is known:** Vosoritide improves growth velocity in children with achondroplasia in controlled clinical trials, which typically exclude patients with prior surgical interventions. Real-world data on vosoritide's effectiveness in clinical practice are limited.

**What is new:** In real-world practice, vosoritide therapy shows significant height and arm span gains even in postsurgical patients, suggesting an early proportional skeletal response, with consistent benefit across varied clinical profiles.

Achondroplasia is the most common form of nonlethal skeletal dysplasia, with a prevalence of approximately 1 in 25 000 live births [1]. It is caused by a gain-of-function mutation in the FGFR3 gene, resulting in impaired endochondral ossification [2]. Clinically, achondroplasia is characterized by disproportionate short stature and multiple complications, including foramen magnum stenosis, obstructive sleep apnea, recurrent otitis media, hearing impairment, spinal stenosis, genu varum, and an increased risk of obesity [3, 4]. At the completion of pubertal growth, patients with achondroplasia attain an average adult height of approximately 131 cm in males and 124 cm in females [5], which contributes to functional limitations and reduced health-related quality of life [6]. Historically, management has focused upon supportive and surgical interventions for associated complications. Recombinant human growth hormone produces only modest and transient effects [7], while limb-lengthening surgery, although effective in increasing stature, is invasive and associated with considerable morbidity [8].

Vosoritide, a C-type natriuretic peptide analog, became the first targeted pharmacologic therapeutic agent for achondroplasia. Following the results from a phase 3 trial demonstrating increased growth velocity (+1.57 cm/year vs placebo) [9], the Food and Drug Administration approved vosoritide in 2020 (initially for patients ≥5 years of age and later expanded in October 2023 to children with open epiphyses of any age), and the European Medicines Agency authorized its use in 2021 (initially ≥2 years and later extended to 4 months of age) [10, 11]. In Israel, vosoritide was approved for reimbursement through the national health basket in 2024, enabling access by eligible patients with open epiphyses. Long-term extension studies have shown sustained benefits, with an average additional cumulative height gain of ∼11 cm after 7 years of continuous treatment [12].

Emerging real-world evidence supports these findings. Recent cohort studies from Portugal, Germany, Italy, and Japan have demonstrated its effectiveness in routine clinical practice [13-16]. Clinical trials, however, typically exclude patients with prior surgical interventions; however, many children with achondroplasia undergo procedures such as foramen magnum decompression for neurological complications or limb lengthening for stature enhancement. Understanding treatment effectiveness across diverse real-world populations strengthens evidence for its broader clinical applicability.

This study presents outcomes of vosoritide therapy in a real-world pediatric cohort with achondroplasia, including those with prior surgical interventions. We evaluated linear growth, sitting height, and arm span in order to characterize both overall growth response and changes in body proportions following treatment.

## Methods

### Study design and participants

This retrospective, single-center cohort study was conducted at the multidisciplinary Achondroplasia Clinic within the Dana-Dwek Children's Hospital, Tel Aviv Sourasky Medical Center, between January 2023 and June 2025. The clinic provides coordinated multidisciplinary care by pediatric endocrinologists, bone health specialists, neurosurgeons, orthopedic surgeons, neurologists, occupational therapists, ear, nose and throat specialists, sleep specialists, pulmonologists, and a psychosocial team.

**Figure 1:**
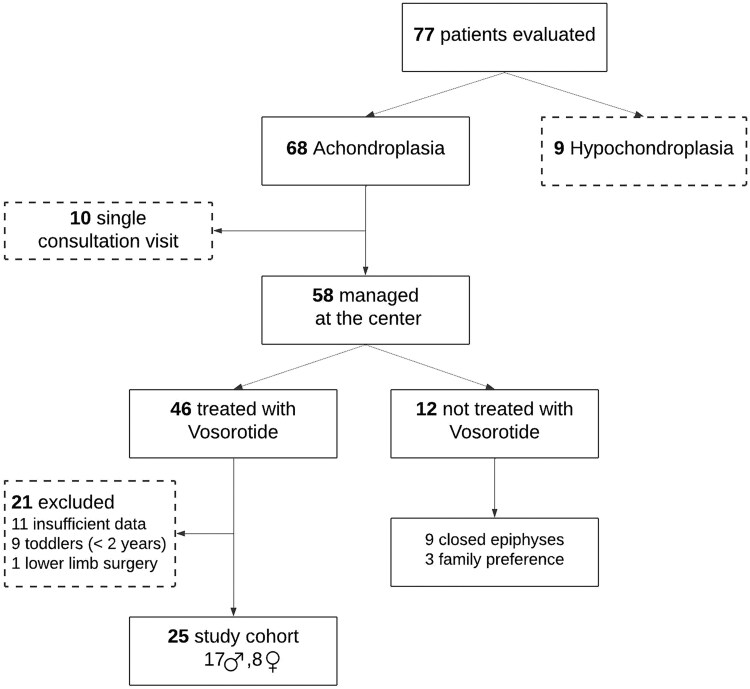
Study population.

During the study period, 77 pediatric patients were assessed in our Achondroplasia Clinic, including 68 with achondroplasia and 9 with hypochondroplasia. Of these, 46 patients received vosoritide treatment, and 22 were not treated. A total of 25 vosoritide-treated patients were suitable for inclusion in the final analysis (Fig. 1). Patients were eligible for study inclusion if they had a confirmed genetic diagnosis of achondroplasia, received vosoritide treatment at our center, and had available anthropometric measurements at both baseline and follow-up. They were excluded if they had hypochondroplasia, closed epiphyses at baseline, incomplete follow-up assessment, missing key anthropometric measurements, were transferred for care to another medical center, or had undergone lower limb surgical intervention during treatment. Nine patients <2 years of age at treatment initiation were excluded due to insufficient follow-up since vosoritide was approved for this age group in Israel only in 2025. Eleven patients (>2 years of age) did not complete the 1-year follow-up due to incomplete treatment, scheduling conflicts, or missed appointments. Five additional patients were excluded from arm span analysis, 1 due to upper limb lengthening surgery and 4 due to incomplete arm span measurements at baseline or follow-up, leaving a total of 20 children for calculating arm span outcomes.

Vosoritide was given as a daily subcutaneous injection at the protocol-recommended dose of 15 μg/kg [10]. As part of the routine standard of care at our center, participants were scheduled for clinic visits at vosoritide initiation, 3 months, 6 months, and every 6 months thereafter. Baseline measurements were defined as the most recent anthropometric assessment obtained within 3 months prior to vosoritide initiation. Follow-up measurements were obtained at least 10 months after starting vosoritide therapy, with a target follow-up of 1 year. This retrospective study was approved by the Institutional Review Board of Dana-Dwek Children's Hospital (Protocol 0001-25-TLV). The requirement for informed consent was waived due to the retrospective nature of the analysis and use of anonymized data.

### Data collection

Anthropometric measurements were obtained by our trained staff during the patients' routine clinic visits and subsequently extracted from electronic medical records. Measurements included height, sitting height, arm span, and weight (measured in light clothing), all obtained by means of calibrated equipment. Height was measured using a Holtain stadiometer (Holtain Ltd., Crosswell, UK) with patients standing barefoot in an upright position, heels and back against the stadiometer, and the head aligned in the Frankfort horizontal plane. Arm span was measured with the patient standing (or seated if needed for stability), arms fully extended horizontally at shoulder level, palms facing forward, and fingers spread. The distance between the tips of the middle fingers of both hands was recorded. Because accurate measurement of linear growth is particularly challenging in children with achondroplasia, 2 measurements were taken, and a third was obtained if the difference exceeded 0.5 cm, with the average of the 2 closest values being used for analysis. Body mass index (BMI) was calculated as weight (kg) divided by height squared (m^2^). Tanner stage assessed at treatment initiation by a Pediatric Endocrinologist was extracted from clinical records. All anthropometric measurements and corresponding z-scores were computed with an AI-assisted growth-monitoring application developed and validated in our clinic, which integrates published achondroplasia-specific lambda mu sigma (LMS) references together with European normative growth standards for automated z-score and percentile calculations [17]. This tool has been shown to improve accuracy and efficiency compared with manual methods.

### Statistical analysis

Descriptive statistics are presented as mean ± standard deviation (SD) for normally distributed continuous variables, and as median with interquartile range (IQR) for non-normally distributed variables. Categorical variables are reported as counts and percentages. Normality was assessed using the Kolmogorov-Smirnov test. Primary pre-post comparisons for changes in anthropometric z-scores were performed using one-sample t-tests for variables with normal distribution or one-sample Wilcoxon signed-rank test for variables with skewed distribution (testing the null hypothesis that the mean change equals zero). Between-group comparisons for categorical variables were performed using Chi-square or Fisher's exact tests, as appropriate. Exploratory analyses examined associations between baseline characteristics (age, sex, BMI z-score, and surgical history) and treatment response using correlation and multivariable backward stepwise linear regression. A 2-sided *P*-value < .05 was considered statistically significant. All analyses were conducted using SPSS version 29.0 (IBM Corp., Armonk, NY, USA).

## Results

### Patient characteristics

Twenty-five children with achondroplasia (17 boys and 8 girls) were included in the analysis. The mean age at vosoritide treatment initiation was 8.3 ± 3.1 years (range 2.9-14.3 years), and the mean follow-up duration was 12.7 ± 1.9 months (range 10-16 months). At treatment initiation, 21 patients (84%) were Tanner stage I, 2 (8%) were Tanner stage II, 1 (4%) was Tanner stage III, and 1 (4%) was Tanner stage IV. Baseline characteristics of the study cohort are summarized in Table 1. Thirteen patients (52%) had undergone foramen magnum decompression, 16 (64%) had undergone a tonsillectomy/adenoidectomy, and 8 (32%) had undergone prior limb-lengthening surgery. The cohort's baseline height z-score was −0.62 ± 1.09 (range −2.76 to +2.05), their sitting height z-score was −1.20 ± 1.12 (range −3.53 to +1.31), their arm span z-score (N = 20) was −1.28 ± 0.93 (range −3.29 to +0.17), and their BMI z-score was −0.64 [−1.38, 0.44].

**Table 1:** Baseline characteristics of 25 children with achondroplasia treated with vosoritide

Characteristic	
Sex, n	Males 17; Females 8
Age at treatment initiation, years	8.3 ± 3.1 (2.9 to 14.3)
Follow-up duration, months	12.7 ± 1.86 (10 to 16)
Height, z-score	−0.62 ± 1.09 (−2.76 to +2.05)
Sitting height, z-score	−1.20 ± 1.12 (−3.53 to +1.31)
Arm span, z-score (n = 20)	−1.28 ± 0.93 (−3.29 to 0.17)
BMI, z-score	−0.64 [−1.38, 0.44]
Foramen magnum decompression, n (%)	13 (52)
Tonsillectomy/adenoidectomy, n (%)	16 (64)
Limb-lengthening surgery, n (%)	8 (32)

Data are presented as mean ± standard deviation and median (IQR) and (number %).

Abbreviation: BMI, body mass index.

### Growth outcomes

**Figure 2:**
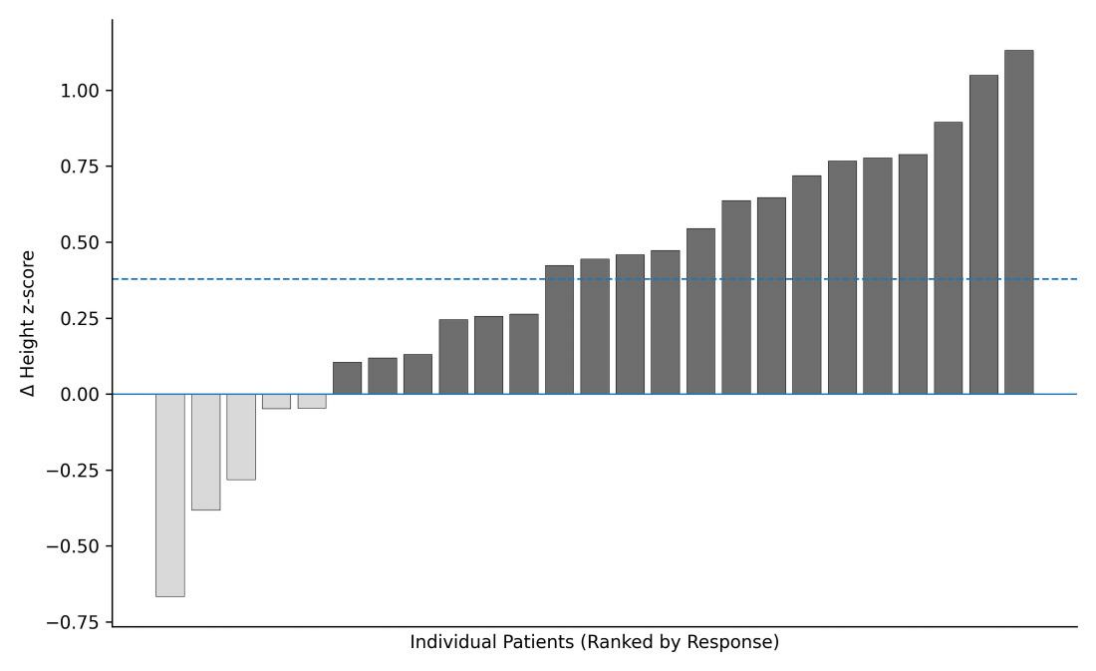
Waterfall plot illustrating the individual change in height z-score from baseline to follow-up during vosoritide treatment. The horizontal dotted line represents the mean Δ height z-score (+0.38, *P* < .001, 1-sample t-test). The dark gray bars indicate an increase in Δ height z-score, and the light gray bars indicate a decrease.

**Figure 3:**
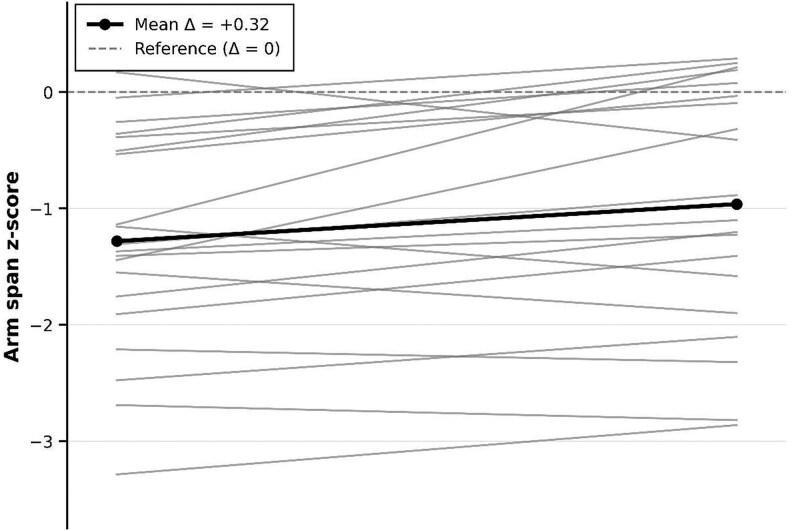
Individual trajectories of arm span z-score changes during vosoritide treatment. Spaghetti plot showing individual Δ arm span z-score from baseline to follow-up (20 patients). Each line represents one patient's trajectory. The horizontal dashed line at 0 represents the reference achondroplasia population mean (Δ = 0). Five patients were excluded (1 had undergone upper-limb lengthening during vosoritide treatment and data were incomplete for 4).

Height z-scores improved from −0.62 ± 1.09 to −0.24 ± 1.20 (*P* < .001) (Table 2). The arm span z-score increased from −1.28 ± 0.93 to −0.96 ± 0.91 (*P* = .007). The sitting height z-scores showed a non-significant trend toward improvement (+0.16 ± 0.53, *P* = .17). BMI z-scores remained stable. The mean Δ height z-score was +0.38. The individual change in height z-score from baseline to follow-up during vosoritide therapy is shown in Fig. 2. Twenty-one patients (84%) demonstrated an increase in Δ height z-score, while 4 patients (16%) showed a decrease. The 4 patients who demonstrated a decrease in height z-score ranged in age from 3.0 to 9.6 years at treatment initiation. None had undergone lower limb surgery during the treatment period. The magnitude of decline was variable (range −0.05 to −0.67 SDS). Arm span responses were discordant in two of these patients, who showed an increase in arm span z-score despite a decline in height z-score. The individual trajectories in arm span z-score from baseline to follow-up during vosoritide therapy are shown in Fig. 3. The mean Δ arm span z-score was +0.32 ± 0.48 (range −0.58 to +1.35). Seventeen patients (85%) demonstrated an increase in arm span z-score, while 3 (15%) underwent a decrease.

**Table 2:** Anthropometric changes in 25 patients with achondroplasia during vosoritide therapy

Characteristic	Baseline mean ± SD	Follow-up mean ± SD	Δ (Follow-up minus baseline)	*P*-value
Height z-score	−0.62 ± 1.09	−0.24 ± 1.20	+0.38 ± 0.45	<.001
Sitting height z-score	−1.20 ± 1.12	−1.04 ± 1.33	+0.16 ± 0.53	.17
Arm span z-score (*n* = 20)	−1.28 ± 0.93	−0.96 ± 0.91	+0.32 ± 0.48	.007
BMI z-score	−0.64 [−1.38, 0.44]	−1.00 [−1.47, 0.51]	−0.04 [−0.37, 0.33]	.84

Data are presented as mean ± standard deviation (SD) and median (IQR).

Abbreviation: BMI, body mass index.

A subgroup of 8 patients had undergone limb-lengthening surgery prior to initiating vosoritide treatment (5 lower-limb procedures and 3 involving both upper and lower limbs). These patients did not significantly differ from the rest of the cohort in age at treatment initiation or baseline height z-score. The mean Δ height z-score increase in this subgroup was comparable to the increase observed in the entire cohort (+0.39 ± 0.36 vs +0.38 ± 0.45, *P* = .97).

### Predictors of growth response

Exploratory analyses that examined potential predictors of response revealed that increases in Δ anthropometric indices (height, sitting height, and arm span) were not significantly associated with sex, age, BMI z-score, or history of surgical interventions. The small sample size, however, limited the strength of these analyses.

### Safety and tolerability

No patients discontinued treatment during the study period. Thirteen patients (52%) reported minor adverse events. All 13 reported injection-site reactions, including transient pain, burning sensation, redness, or swelling at the injection site, all of which resolved spontaneously without dose modification. One patient (4%) reported a single episode of dizziness. One patient (4%) reported hirsutism during the treatment period; a causal relationship to vosoritide could not be established. No serious treatment-related adverse events occurred. All exclusions from the final analysis were due to incomplete anthropometric data and were not related to adverse events or treatment tolerability.

## Discussion

In this real-world pediatric cohort, vosoritide treatment was associated with improvement in linear growth, accompanied by gains in appendicular length. The growth response observed in our cohort aligns with results from clinical trials and early post-approval studies [9, 13-16] and extends current knowledge by including patients across a broader age spectrum and those with prior neurosurgical and orthopedic procedures who commonly present in practice but are under-represented in trial settings.

The magnitude of height z-score improvement in our study (+0.38) was approximately 40% lower than the +0.61 z-score change reported in Savarirayan et al's pivotal randomized controlled trial at 52 weeks [9]. Rather than indicating reduced efficacy, this difference reflects the expected effect when treating real-world populations: our cohort included older children and adolescents approaching epiphyseal closure, and, importantly, 32% of them had undergone prior surgical interventions, representing an extension of populations from other trials. Our findings align with emerging real-world evidence from European and Asian cohorts that report 12-month outcomes, with all demonstrating similar height z-score gains of +0.35 to +0.45, suggesting that vosoritide's effectiveness is reproducible across diverse clinical settings [14-16]. Importantly, our findings exceeded the expected trajectory from natural history studies of untreated children with achondroplasia, confirming that vosoritide provides growth acceleration in routine practice.

Non-responders have also been documented in other real-world cohorts; Wechsung et al reported that 7.6% of 165 children in a multicenter European registry showed a height SDS decrease at 12 months, with the proportion halving by year 2, suggesting some patients demonstrate delayed response [18]. In real-world cohorts, variability in growth response can occur and may reflect differences in baseline growth potential and individual biological variability.

Studies from Germany (Reincke et al, 2025) [15], Italy (Allegri et al, 2025) [16], and Japan (Kitoh et al, 2025) [14], all using the European achondroplasia-specific Merker et al (2018) [19] LMS growth charts, reported mean height z-score increases ranging from +0.35 to +0.45 after 12 months of treatment, closely mirroring the changes observed in our cohort. In contrast, a very recent Portuguese study by Rua et al [13], which applied the U.S. achondroplasia reference charts from Horton et al (2007) [1] and assessed outcomes over a longer (18 to 24 months) period, recorded a larger cumulative z-score gain. These differences likely reflect both the extended follow-up and the use of different growth charting systems. When assessed using the Merker LMS references, our results align with the European and Japanese data, underscoring the reproducibility of vosoritide's growth effects across diverse clinical settings.

Our finding that limb-lengthening surgery did not seem to modify the growth response to vosoritide aligns with results from the Japanese multicenter study by Kitoh et al (2025) [14], which included 26 children with achondroplasia treated for at least 12 months. In that cohort, the mean height z-score increased by +0.4, with no differences between patients who had undergone limb lengthening and those who had not. Collectively, these results support the notion that vosoritide remains effective in promoting growth, regardless of prior limb-lengthening surgery. When interpreting these observations, however, it should be borne in mind that both the Japanese and our studies included only small cohorts.

Body proportion is a key clinical focus in patients with skeletal dysplasia, given its implications for function, mobility, and overall quality of life. In our cohort, baseline arm span z-scores were markedly lower than height z-scores, suggesting either greater variability or more pronounced upper extremity involvement. The comparable gains in arm span and height observed after treatment with vosoritide suggest an early proportional skeletal response and indicate that vosoritide may help preserve or improve body proportion despite initial limb–trunk discrepancy. Several interpretations of this finding merit consideration. First, the treatment interval may be insufficient to detect meaningful changes in axial growth, given that proportional trunk contributions to body proportion might emerge more slowly than appendicular gains. Second, the use of sitting height to assess trunk length is inherently more prone to technical variability than standing height or arm span, potentially obscuring subtle but clinically relevant changes. Third, the findings may reflect a true physiological pattern, with vosoritide preferentially enhancing appendicular skeletal elements during the early treatment phase. Longer-term studies, however, have provided mixed insights into the evolution of body proportion under therapy. The Portuguese cohort's findings suggest that improvements in trunk-limb proportionality may not readily emerge within the first 2 years of treatment, raising the possibility that axial skeletal response to vosoritide could be delayed or less pronounced over this timeframe [13]. Conversely, the 2-year open-label extension of the phase 3 trial reported gradual improvements in proportionality indices, implying delayed axial growth [12]. These discrepancies may result from variations in methodology, participant characteristics, or analytical approaches.

Obesity and weight gain are well-recognized concerns in achondroplasia [20, 21]. The wide variability in BMI z-score changes observed in our cohort (ranging from −0.91 to +1.92) highlights the need for individualized nutritional monitoring. Accordingly, systematic weight surveillance, nutritional assessment, and dietary counseling should be integrated into routine care for children receiving vosoritide.

### Strengths and limitations

This study provides real-world evidence on the response to vosoritide in a diverse pediatric achondroplasia cohort, including patients with prior surgical interventions and a wide age range reflective of routine clinical practice. Standardized anthropometric assessments and growth trajectory evaluation strengthened the interpretation of longitudinal changes. The inclusion of segmental growth measures, specifically, arm span, added to the limited evidence base on appendicular growth in real-world settings. The use of a validated AI-assisted application for automated z-score calculation enhanced precision and efficiency [17].

This analysis is limited by its retrospective design and the relatively small sample size inherent to a rare condition. In addition, although follow-up was planned at 3- to 6-month intervals, the timing of clinic visits depended on patient availability, reflecting real-world practice rather than a strict protocol. Since the duration of follow-up was restricted to the first treatment year, long-term effects on growth and body proportions could not be assessed.

Longitudinal follow-up of this cohort will be essential to assess sustained growth response, proportionality, quality of life outcomes, and functional parameters, and determine whether anthropometric improvements translate into meaningful patient-centered benefits. Comparative data across centers, including initiation of treatment at younger ages and in combination with multidisciplinary management, will enhance evidence-based care and help guide individualized therapeutic decision-making in achondroplasia.

## Conclusion

In this real-world clinical setting, vosoritide treatment was associated with improved linear and appendicular growth during the first treatment year. Growth response was observed across a broad clinical spectrum, including among patients with prior surgical interventions. Continued longitudinal data collection will be important to determine long-term effects on growth trajectories, skeletal proportions, and overall health outcomes in children with achondroplasia.

## Data Availability

Some or all datasets generated during and/or analyzed during the current study are not publicly available but are available from the corresponding author on reasonable request.
